# The effect and safety of obeticholic acid for patients with nonalcoholic steatohepatitis: A systematic review and meta-analysis of randomized controlled trials

**DOI:** 10.1097/MD.0000000000037271

**Published:** 2024-02-16

**Authors:** Jie Zhao, Baozhen Li, Kai Zhang, Zhiyong Zhu

**Affiliations:** a Department of Nephrology, Zibo Central Hospital, Zibo, China; b Department of Gastroenterology, Zibo Central Hospital, Zibo, China; c Shandong Drug and Food Vocational College, Weihai, China.

**Keywords:** dyslipidemia, meta-analysis, NASH, obeticholic acid, pruritus

## Abstract

**Background and aims:**

Nonalcoholic fatty liver disease/nonalcoholic steatohepatitis (NASH) is one of the primary causes of chronic liver disease worldwide. Obeticholic acid (OCA), a potent farnesoid X nuclear receptor activator, has shown promise for treating NASH-related fibrosis due to its anti-fibrotic effects. This study aimed to examine the efficacy of OCA for patients with NASH as well as to investigate its impact on dyslipidemia.

**Method:**

A search of databases including PubMed, Embase, and Cochrane Library from January 1, 2010, to November 1, 2022, was conducted to identify systematic reviews of randomized controlled trials involving NASH patients. Inclusion criteria comprised randomized controlled trials that specifically addressed NASH as diagnosed through magnetic resonance imaging, computed tomography, or histology. The results were then categorized, with consideration given to both biochemical and histological outcomes.

**Result:**

Five NASH studies were ultimately selected for further analysis. In terms of biochemical indicators, patients receiving OCA treatment showed improvements in alanine transaminase (mean difference: −19.48, 95% confidence interval [CI]: −24.39 to 14.58; *P* < .05) and aspartate aminotransferase (mean difference: −9.22, 95% CI: −12.70 to 5.74; *P* < .05). As for histological improvement, OCA treatment reduced fibrosis (odds ratio [OR]: 1.95, 95% CI: 1.47–2.59; *P* = .001) and steatosis (OR: 1.95, 95% CI: 1.47–2.59; *P* = .001). No significant differences were observed regarding adverse events (1.44, 95% CI: 0.57–3.62; *P* > .001). Regarding dyslipidemia, mean differences between total cholesterol and low-density lipoprotein were found to be high (0.33, 95% CI: 0.01–0.64, *P* < .05; 0.39, 95% CI: 0.04–0.73, *P* < .05). In the case of pruritus, OCA achieved a high OR (3.22, 95% CI: 2.22–4.74) compared with placebo.

**Conclusion:**

OCA also reduced several liver test markers compared to placebo, including the biochemical indicators alanine transaminase, aspartate aminotransferase, alkaline phosphatase, and γ-glutamyl transpeptidase, and improved hepatocellular ballooning, fibrosis, steatosis, and lobular inflammation. Although the incidence of adverse events did not significantly differ between OCA and placebo groups among NASH patients, OCA treatment was found to elevate total cholesterol and low-density lipoprotein levels, and the reported severity of pruritus increased with higher doses of OCA.

## 1. Introduction

Globally, nonalcoholic fatty liver disease/nonalcoholic steatohepatitis (NAFLD/NASH) is a primary factor contributing to chronic liver disease.^[1]^ NASH may also progress to cirrhosis and related complications, including hepatocellular carcinoma (HCC).^[2]^ NASH, also known as nonalcoholic steatohepatitis, refers to nonalcoholic steatosis with hepatocellular ballooning, inflammation, and fibrosis.^[3]^ Compared to nonalcoholic steatosis patients, NASH patients have a higher mortality rate and cardiovascular disease risk^[4]^ and NASH may lead to liver cancer, which, like cirrhosis, may require liver transplantation.^[5]^ Moreover, NASH is expected to become the primary cause of liver transplantation. Contributing factors to NAFL/NASH prevalence include age, obesity, and type 2 diabetes mellitus, as well as other demographic aspects including country of residence and ethnicity.^[6]^

While lifestyle interventions such as the Mediterranean diet and weight loss are considered positive approaches for managing NASH,^[7]^ they do not usually reverse the fibrosis associated with the condition. Regulatory approval by the US Food and Drug Administration (FDA) for therapies addressing NASH-related fibrosis must demonstrate their effectiveness in improving long-term clinical outcomes, and despite the accumulation of a large volume of research on the pathogenesis of NASH over the past decade, no approved treatment currently exists.^[8]^ However, Phase 2b and 3 data from some studies suggest that semaglutide, liraglutide, and vitamin E may be more effective than placebo.^[9–11]^

The bile acid 6-ethyl-chenodeoxycholic acid (obeticholic acid, or OCA), is a synthetic version of chenodeoxycholic acid and serves as a potent farnesoid X nuclear receptor (FXR) activator.^[12]^ Initially approved by the FDA to treat primary biliary cholangitis,^[12]^ OCA gained attention in 2016 when a study suggested that OCA treatment has the potential to lower NASH patients’ NAFLD activity scores (NAS), and improve fibrosis, steatosis, hepatic lobule inflammation, and hepatocellular ballooning, which may reverse the severity of NASH-related liver fibrosis. However, the study also reported severe pruritus as a side effect, limiting the widespread application of OCA.^[13]^

While several network meta-analyses have evaluated OCA treatment for liver fibrosis,^[14,15]^ no meta-analysis has specifically focused on the effects of OCA in NASH patients. Furthermore, there have been no quantitative analyses of side effects and no relevant meta-analyses investigating OCA’s impact on blood lipids, especially in patients with NASH. Therefore, this meta-analysis aimed to comprehensively assess the impact and safety of OCA treatment for NASH patients.

## 2. Methods

In this study, a systematic review and network meta-analysis were conducted on prior studies assessing the effectiveness of OCA in treating NASH. All reported findings adhered to the Preferred Reporting Items for Systematic Reviews and Meta-Analyses (PRISMA) guidelines. Ethical approval was not required, as all data were obtained from prior research.

### 2.1. Search strategy

The PubMed, Embase, Web of Science, and Cochrane databases were searched to identify studies conducted between January 1, 2010, and November 1, 2022. Only English language studies were considered for analysis.

The search strategy utilized keywords such as OCA, obeticholic acid, nonalcoholic steatohepatitis, nonalcoholic fatty liver disease, NASH, and NAFLD. Additional details on the search strategies can be found in Table S1, Supplemental Digital Content, http://links.lww.com/MD/L651.

### 2.2. Inclusion and exclusion criteria

The analysis included studies meeting the following predefined criteria: (1) an randomized controlled trial (RCT); (2) NASH diagnosed by magnetic resonance imaging, computed tomography, or histology; and (3) biochemical and histological outcomes were measured. Abstracts, reviews, case reports, and letters were not included. Studies that did not demonstrate an effect from OCA on NASH patients were also excluded.

### 2.3. Study selection process

Two independent investigators (Zhao, J. and Li, B.Z.) reviewed the titles and abstracts of all studies identified by the search process and independently screened the included abstracts of full-text manuscripts for eligibility. Disagreements were resolved through consultation with a third reviewer (K Zhang) when necessary to reach a consensus.

### 2.4. Data extraction and risk of bias assessment

Data extraction for this study was performed by Zhao, J. and Li, B.Z., utilizing a standardized form, which included the following information: (a) details about the included studies (first author, publication year, location, and duration of follow-up); (b) biochemical outcomes, including alanine transaminase (ALT), aspartate aminotransferase (AST), γ-glutamyl transpeptidase (GGT), and alkaline phosphatase ALP); (c) pathological results; (d) adverse events.

To assess for risk of bias in the RCTs, the Cochrane Risk of Bias assessment tool in the Review Manager 5.4.1 software was utilized.

### 2.5. Data synthesis and statistical analysis

STATA 15.1 software (Stata Corporation, College Station, TX) was employed for data analysis. Dichotomous variables were expressed as odds ratios (ORs) to indicate their effectiveness using a 95% confidence interval (CI). Quantitative variables were presented as mean differences (MDs) (averages plus standard deviations), i.e., the difference between mean values following treatment. Heterogeneity was evaluated using the Q statistic, with *I*^2^ > 50% indicating heterogeneity. In the absence of literature heterogeneity, a fixed-effect model was employed; otherwise, a random-effect model was applied. Subgroup analysis/meta-regression and sensitivity analyses were used when *I*^2^ exceeded 50% to further analyze instances of high heterogeneity in the data.

## 3. Results

### 3.1. Results of the study search

**Figure 1.**
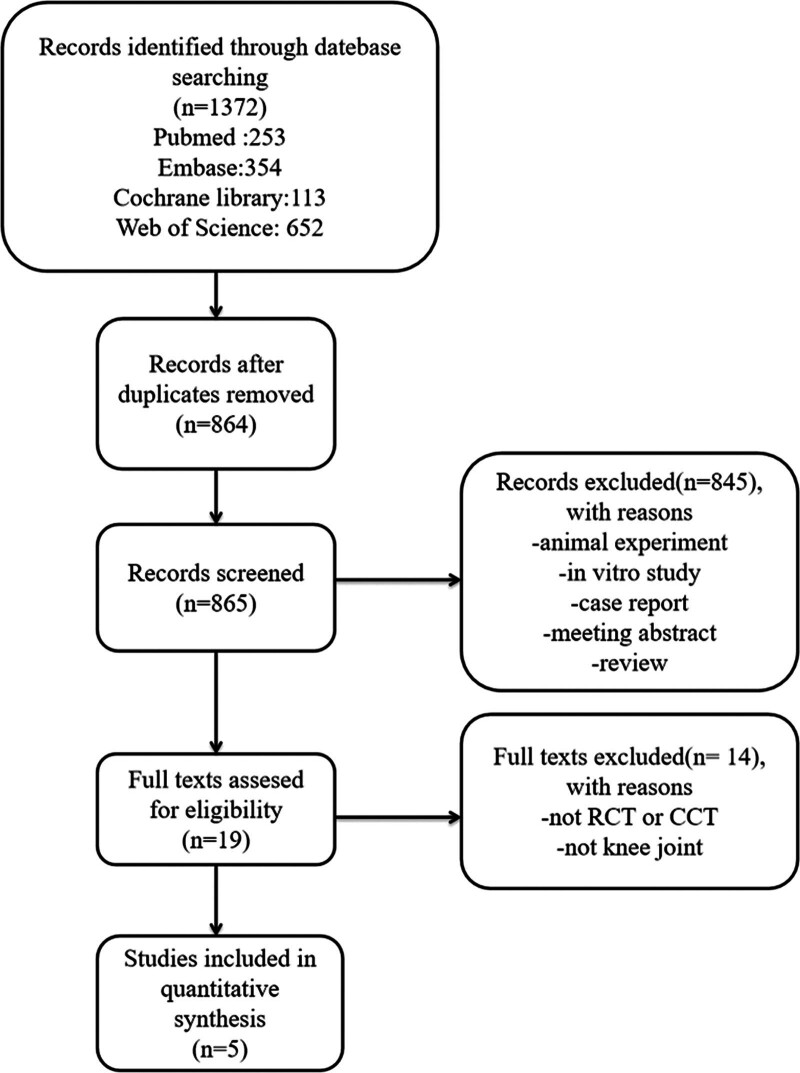
Flowchart of study selection.

A total of 1372 records were identified by the search strategy. All references were then imported into the reference management software. An overview of the systematic review and meta-analysis process can be seen in the flowchart (Fig. 1). Ultimately, 5 studies were included for analysis.^[13,16–19]^

### 3.2. Baseline characteristics and quality assessment

**Figure 2.**
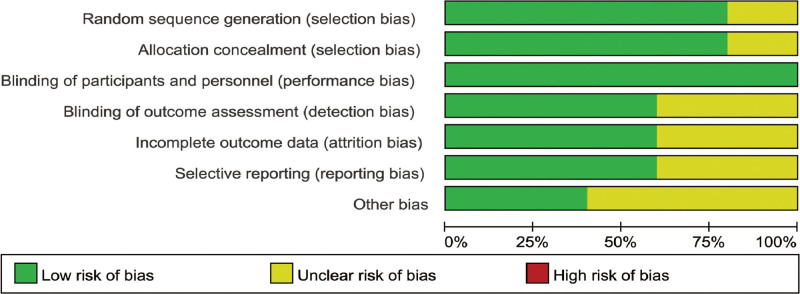
Risk of bias graph.

In total, data from 2336 participants were involved in our analysis. All studies included were published within the past decade (2013–2022) and were conducted in the United States. A comprehensive overview of the baseline characteristics of the included trials is presented in Table 1. Review Manager 5.4.1 was used to evaluate the risk of bias in the RCTs. The risk of bias model is shown in Figure 2.

**Table 1:** Baseline characteristics of included studies.

Author	Year	Country	Design	N	Intervention	Duration	NASH diagnosis
Mary E. Rinella	2022	USA	RCT	931	OCA 10 mg; OCA 25 mg	18 months	Pathology
Paul J. Pockros^[19]^	2019	USA	RCT	49	OCA 5 mg; OCA 10 mg; OCA 25 mg	6 weeks	Imaging or pathology
Zobair M Younossi^[17]^	2019	USA	RCT	931	OCA 10 mg; OCA 25 mg	18 months	Pathology
Brent A Neuschwander-Tetri^[13]^	2015	USA	RCT	283	OCA	24 months	Pathology
Sunder Mudaliar^[18]^	2013	USA	RCT	64	OCA 25 mg; OCA 50 mg	6 weeks	Imaging or pathology

OCA = obeticholic acid, RCT = randomized controlled trial.

### 3.3. Biochemical indicators improvement in NASH

**Figure 3.**
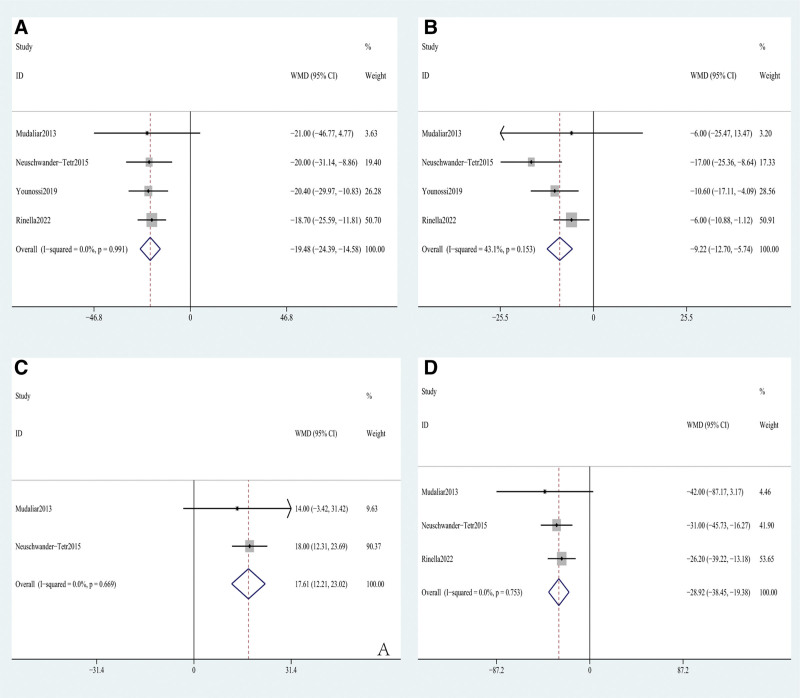
Biochemical indicators improvement in NASH. (A) ALT; (B) AST; (C) ALP; (D) GGT. ALP = alkaline phosphatase; ALT = alanine transaminase; AST = aspartate aminotransferase; GGT = γ-glutamyl transpeptidase.

The results of our study show that OCA contributes to a reduction in biochemical indicators, including ALT, AST, ALP, and GGT (Fig. 3). NASH patients receiving OCA treatment showed improvements in ALT (MD: −19.48, 95% CI: −24.39 to 14.58; *P* < .05) and AST (MD: −9.22, 95% CI: −12.70 to 5.74; *P* < .05) compared to NASH patients who received a placebo. Regarding ALP, an MD of 17.61 (95% CI, 12.21–23.02; *P* < .05) was observed, also indicating an improvement. Additionally, a difference was identified between OCA treatment and placebo for GGT levels (MD: −28.92, 95% CI, −38.45 to 19.38; *P* < .05).

### 3.4. Histological improvement in NASH

**Figure 4.**
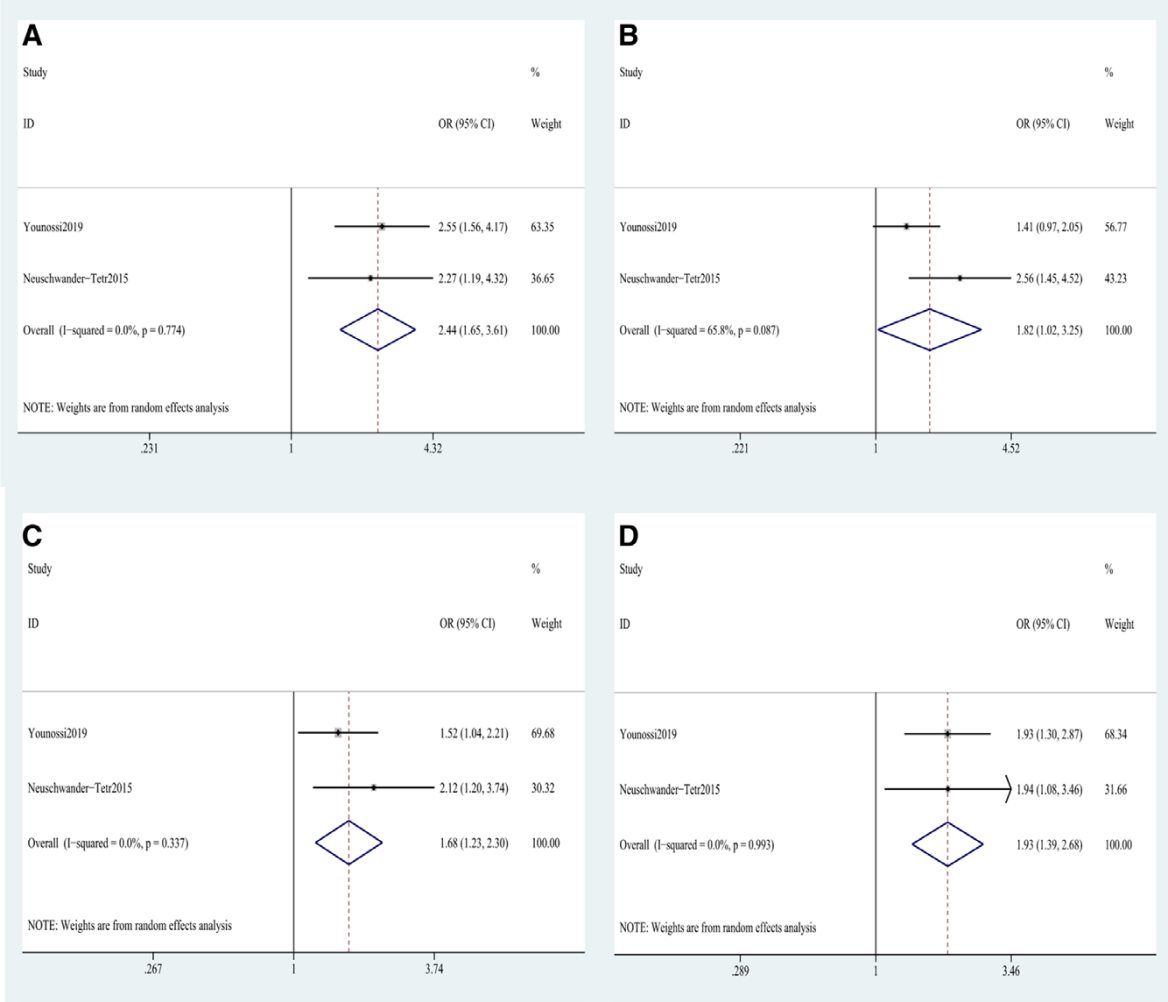
Histological improvement in NASH. (A) fibrosis; (B) lobular inflammation; (C) lobular inflammation; (D) hepatocellular ballooning. NASH = nonalcoholic steatohepati.

Our findings also reveal that OCA treatment affects noticeable histological improvements for fibrosis, steatosis, lobular inflammation, and hepatocellular ballooning, compared to placebo (Fig. 4). Greater reductions in fibrosis (OR: 2.44, 95% CI: 1.65–3.61; *P* = .001) and steatosis (OR: 1.82, 95% CI: 1.02–3.65; *P* = .001) were observed in NASH patients receiving OCA treatment compared to NASH patients receiving placebo. Lobular inflammation similarly improved in NASH patients, (OR:1.68, 95% CI: 1.23–2.30; *P* = .001; *I*^2^ = 0, *P* = .337). Furthermore, differences were found between OCA treatment and placebo groups regarding their degree of hepatocellular ballooning (OR: 1.93, 95% CI: 1.39–2.68; *P* = .001).

### 3.5. The safety of OCA

As for adverse events (AEs), no significant difference (1.44, 95% CI:0.57–3.62; *P* > .001) was found between NASH patients who received OCA treatment compared to those who received a placebo (Table 2). However, with regard to pruritus, OCA exhibited a high OR of 3.22 (95% CI: 2.22–4.74) compared to placebo. Furthermore, the 25 mg OCA groups showed higher odds of pruritus than the 10 mg OCA groups (OR: 4.72, 95% CI: 3.41–6.52, *P* < .05; 1.68, 95% CI: 1.30–2.18, *P* < .05), indicating that higher doses of OCA are associated with more severe pruritus.

**Table 2:** The adverse events of OCA.

	OR(95%CI)	*P*	*I* ^2^	*P*
AEs	1.44 (0.57–3.62)	*P* > .05	57.1%	*P* < .05
Pruritus				
OCA vs placebo	3.22 (2.22–4.74)	*P* < .05	19.3%	*P* > .05
10 mg OCA vs placebo	1.68 (1.30–2.18)	*P* < .05	0.0%	*P* > .05
25 mg OCA vs placebo	4.72 (3.41–6.52)	*P* < .05	54.9%	*P* < .05

AEs = adverse events, OCA = obeticholic acid.

Regarding dyslipidemia, total cholesterol (TC) and low-density lipoprotein (LDL) levels exhibited high mean differences (0.33, 95% CI: 0.01–0.64, *P* < .05; 0.39, 95% CI: 0.04-0.73, *P* < .05) among OCA treatment groups compared to those who received a placebo (Table 3). Nevertheless, high-density lipoprotein and triglyceride levels of NASH patients receiving OCA did not significantly differ from the placebo groups (MD: −0.19, 95% CI: −0.18 to 0.00; *P* > .05 and −0.06, 95% CI: −0.52 to 0.4; *P* > .05, respectively).

**Table 3:** The dyslipidemia of OCA.

	MD (mean ± sd)	*P*	*I* ^2^	*P*
TC	0.33 (0.01–0.64)	<.05	0.0%	>.05
LDL	0.39 (0.04–0.73)	<.05	0.0%	>.05
HDL	−0.19 (−0.18–0.00)	>.05	0.0%	>.05
TG	−0.06 (−0.52–0.4)	>.05	0.0%	>.05

HDL = high-density lipoprotein cholesterol, LDL = low-density lipoprotein cholesterol, TC = total cholesterol, TG = triglycerides.

## 4. Discussion

This systematic review and meta-analysis evaluated the efficacy of OCA for the treatment of NASH. Our findings indicate that NASH patients treated with OCA experienced statistically significant reductions in biochemical indicators such as ALT, AST, ALP, and GGT, compared to those treated with a placebo. Moreover, OCA treatment resulted in substantial improvements in histological conditions such as fibrosis, steatosis, lobular inflammation, and hepatocellular ballooning. With the exception of pruritus, OCA did not appear to significantly contribute to AEs compared to placebo, with pruritus severity increasing at higher OCA doses. Additionally, higher levels of TC and LDL were also observed in the OCA treatment group.

The therapeutic functions contributing to OCA’s efficacy for NASH patients has been explored in several studies. As a farnesoid X receptor agonist, OCA exerts a strong effect on this receptor.^[20,21]^ OCA binds to and activates FXR, leading to increases in fibroblast growth factor-19 (FGF19) secretions by the ileum. Activation of FGF19 reduces bile acid synthesis, potentially mitigating lipid dysregulation in NASH patients.^[22]^ OCA also inhibits the transforming growth factor-β gene, influences extracellular matrix reorganization, and suppresses hepatic stellate cell activation. FXR agonism with OCA has been shown to lead to a reduction in monocyte chemoattractant protein-1 mRNA expression in NASH models, decreasing inflammatory cell infiltration and further improving fibrosis.^[23]^ In summary, OCA exhibits antifibrotic, anti-cholestatic, and anti-inflammatory properties.^[16]^

Several studies have previously evaluated the efficacy of OCA treatment for liver fibrosis, but these studies,^[14,15,24,25]^ were comprised of just two or three trials and only evaluated liver fibrosis indicators. Importantly, prior research has neglected to perform either a quantitative analysis to explore side effects or a meta-analysis to assess the impact of OCA on blood lipids, especially for NASH patients. Therefore, further research is required to evaluate the therapeutic effects and detail the possible side effects of OCA across various indicators to enhance drug development and clinical applications. This study, by aggregating biochemical and histological indicators of NASH improvement as well as identifying instances of AEs, offers valuable insight for clinical practitioners. Additionally, we provided a detailed summary of OCA’s effects on pruritus and dyslipidemia, which significantly impact NASH patients’ quality of life.

Given the pivotal role of fibrosis in the progression of NASH, anti-fibrosis treatment is essential.^[26]^ OCA, through its inhibition of the transforming growth factor gene,^[27]^currently remains the only well-recognized approach for alleviating NASH-related fibrosis. In our meta-analysis, OCA demonstrated a significant improvement in fibrosis without worsening NASH symptoms with an OR of 2.44 (95% CI: 1.65–3.61). This effect may be compared with PRI-724, a novel drug that inhibits hepatic stellate cell activation through the Wnt/β-catenin signaling pathway. In the future, the development of additional hepatic stellate cell activation inhibitors promises to provide more effective treatments for NASH patients.^[28]^

Based on our meta-analysis, OCA did not significantly differ from placebo in terms of AEs (OR: 1.44, [0.57–3.62]; *P* > .001). Nevertheless, some notable disadvantages of OCA treatment were identified, including the potential development of pruritus and dyslipidemia. The current understanding of pruritus is primarily derived from studies by Younssi, et al and Pockros, et al which compared the prevalence of pruritus among NASH patients in OCA treatment and placebo groups.^[19]^ These studies revealed that patients receiving 25 mg of OCA were more likely to report pruritus than those in placebo groups (4.72 [3.41–6.52], *P* < .05; 1.68 [1.30–2.18], *P* < .05), suggesting that high doses of OCA are associated with pruritus. Despite this finding, pruritus’ underlying pathology remains elusive in many cases.

Several studies have also been conducted on dyslipidemia in NASH patients. Our own research further confirms OCA’s associations with dyslipidemia and increases in TC and LDL levels. Nevertheless, it should be noted that NASH patients receiving OCA exhibited no significant differences in high-density lipoprotein or triglyceride. Furthermore, although OCA appears to reduce bile acid synthesis through the increased secretion of FGF19, the process is still not well understood. Future discussions should consider whether OCA may be used in conjunction with lipid-related drugs to address these effects.

At this juncture, it is worth considering NASH patients’ quality of life. A recent study found that patients treated with OCA who experienced improvements in fibrosis, decreased activity scores for nonalcoholic fatty liver disease, and NASH resolution had better patient-reported outcomes.^[29]^ However, approximately 1.6% of patients discontinued therapy due to grade 3 pruritus, experiencing a worsening of itch symptoms during the first month of treatment.^[29]^ As can be seen, pruritus is a significant factor influencing the quality of life for individuals receiving OCA therapy. Our findings also determined that pruritus tends to be mild and mostly occurs early in the course of OCA treatment. Nevertheless, additional studies are essential if the FDA is going to approve OCA for widespread treatment.

The cost-effectiveness of treating nonalcoholic steatohepatitis with OCA also demands further investigation. A study by Wong et al,^[30]^ which employed a state-transition model, determined that OCA was not cost-effective compared with placebo despite its clinical benefits due to the drug’s high cost. Importantly, the model used in their study only considered OCA at the 25 mg dose in comparison with a placebo. Further research is needed to determine whether other doses, such as 10 mg or lower, may be more cost-effective.

According to a recent meta-analysis, bariatric surgery is associated with a lower risk of hepatocellular carcinoma compared to other types of surgery.^[31]^ OCA may also have the potential to reduce the risk of liver cancer among patients with NASH. One study demonstrated that OCA suppressed activity in the SOCS3/Jak2/STAT3 pathway, mitigating NASH-related HCC development and progression. This finding suggests that FXR activators such as OCA could be employed in the treatment of NASH-related disorders, even in the later stages, as a preventative measure to prevent the progression to HCC.^[32]^ Although there are currently no clinical trials exploring this possibility, the potential therapeutic role of FXR activators warrants further research.

### 4.1. Limitation

The primary limitation of our meta-analysis lies in the small number of studies available. While all included studies were found to be of high quality, only 2 explored OCA’s anti-fibrosis effects, limiting the generalizability of the results. Additionally, the included studies lacked detailed descriptions of adverse reactions, further limiting our attempts at comprehensive analysis. Furthermore, relatively few studies have explored OCA’s effects on cardiovascular-related events, a result of the adverse lipid changes associated with OCA. Addressing these limitations in the future will require extensive clinical trials involving a larger number of patients.

## 5. Conclusion

Our meta-analysis comparing NASH patients receiving OCA with placebo groups reveals several important findings. First, OCA significantly reduces biochemical indicators such as ALT, AST, ALP, and GGT. Additionally, it substantially improves several NASH-related disorders, including lobular inflammation, fibrosis, steatosis, and hepatocellular ballooning. These advantages appear to occur without an increased risk of AEs. The study also identified associations between higher OCA doses and the increased severity of pruritus, as well as effects on TC and LDL levels.

In summary, our meta-analysis underscores the potential therapeutic benefits of OCA in addressing the biochemical and histological aspects of NASH. Further research is imperative to refine our understanding of OCA’s efficacy and safety, as well as its broader impact on quality of life for patients with NASH.

## Author contributions

**Conceptualization:** Zhiyong Zhu, Kai Zhang.

**Data curation:** Zhiyong Zhu, Jie Zhao, Kai Zhang.

**Formal analysis:** Zhiyong Zhu, Jie Zhao, Kai Zhang.

**Funding acquisition:** Zhiyong Zhu, Jie Zhao.

**Investigation:** Zhiyong Zhu, Jie Zhao.

**Methodology:** Jie Zhao.

**Project administration:** Jie Zhao.

**Resources:** Jie Zhao.

**Software:** Jie Zhao, Baozhen Li.

**Supervision:** Jie Zhao, Baozhen Li.

**Validation:** Baozhen Li.

**Visualization:** Baozhen Li.

**Writing – review & editing:** Jie Zhao.

## Supplementary Material


