# Supplementary material for: The effect and safety of obeticholic acid for patients with nonalcoholic steatohepatitis: A systematic review and meta-analysis of randomized controlled trials
Source: Medicine (Baltimore). 2024 Feb 16;103(7):e37271. doi: 10.1097/MD.0000000000037271 (PMC10869096; doi:10.1097/MD.0000000000037271)
Supplement: Supplementary file 1 [file medi-103-e37271-s001.docx]

Table S1 Search strategies

| **Electronic databases** | **Search strategies** |
| --- | --- |
| Cochrane Library | #1 (OCA) OR ('obeticholic acid')  #2 ('nonalcoholic steatohepatitis') OR (NASH) OR ('nonalcoholic fatty liver') OR (NAFLD)  #1 AND #2 |
| Embase | #1 ('nonalcoholic steatohepatitis'/exp OR 'nonalcoholic steatohepatitis') AND 'nonalcoholic fatty liver'  #2 oca OR 'obeticholic acid'  #1 AND #2 |
| Pubmed | #1 (((NASH) OR (Non-Alcoholic Fatty Liver Disease)) OR (Non-Alcoholic Fatty Liver Disease)) OR (NAFLD)  #2 (OCA) OR (Obeticholic acid)  #1 AND #2 |
| Web of Science | #1 TS=("NASH" OR "Non-Alcoholic Fatty Liver Disease" OR "NAFLD" OR "Non-Alcoholic Fatty Liver Disease")  #2 TS=("OCA" OR "Obeticholic acid")  #1 AND #2 |
